# Agarwood oil nanoemulsion attenuates production of lipopolysaccharide (LPS)-induced proinflammatory cytokines, IL-6 and IL-8 in human bronchial epithelial cells

**DOI:** 10.17179/excli2023-6282

**Published:** 2023-07-20

**Authors:** Juman Mohammed Rasmi Alamil, Dikaia Xenaki, Bikash Manandhar, Keshav Raj Paudel, Philip Michael Hansbro, Brian Gregory Oliver, Dinesh Kumar Chellappan, Kamal Dua

**Affiliations:** 1 School of Postgraduate Studies, International Medical University (IMU), Kuala Lumpur 57000, Malaysia; 2 Woolcock Institute of Medical Research, Macquarie University, Sydney, NSW 2137, Australia; 3 Discipline of Pharmacy, Graduate School of Health, University of Technology Sydney, Sydney, NSW 2007, Australia; 4 Australian Research Center in Complementary and Integrative Medicine, Faculty of Health, University of Technology Sydney, Ultimo, NSW 2007, Australia; 5 Center for Inflammation, Centenary Institute and University of Technology Sydney, Faculty of Science, School of Life Sciences, Sydney, NSW 2050, Australia; 6 School of Life Sciences, Faculty of Science, University of Technology Sydney, Ultimo, NSW 2007, Australia; 7 Department of Life Sciences, School of Pharmacy, International Medical University, Bukit Jalil, Kuala Lumpur 57000, Malaysia; 8 Uttaranchal Institute of Pharmaceutical Sciences, Uttaranchal University, Dehradun 248007, India

## ⁯⁯

Inflammation is a protective bodily response towards a variety of insults to different systems or organs. In both asthma and chronic obstructive pulmonary disease (COPD), inflammation begins with the recruitment of immune cells, including lymphocytes, mast cells, macrophages, neutrophils, and eosinophils. These, in turn, produce large amount of pro-inflammatory mediators such as interleukin (IL)- 6, 8, 33, 13, and tumor necrosis factor (TNF)-α that contribute to the inflammatory process (Rovina et al., 2013[7]). The inflammatory cytokines IL-6 and 8 play an important role in the pathogeneses of the disease, where they help in the recruitment and the adhesion of pro-inflammatory cells in the airways leading to the formation of mucus, which is why they have been targeted as promising biomarkers for diagnosis and future therapies. 

The current treatments like long-acting beta-agonists for chronic respiratory diseases, including asthma and COPD, despite being effective in acute exacerbations, have demonstrated limited effect in many chronic cases. Herbal medicines have been under the spotlight lately and have been extensively studied in the past few decades as a newer effective therapy with less side effects compared to conventional chemical drugs. 

Agarwood oil is a resinous oil formed in certain species of trees that grow in areas of southeast Asia and China after getting infected with fungus. Agarwood is one of the promising herbal therapies that has been under study for its medicinal properties and has been found to contain many active compounds that play a role in limiting inflammation. Many *in vivo* and *in vitro* studies have been done in that regard to test the functionality of agarwood extract as an anti-inflammatory agent, and majority of them have showed encouraging results. For example, in a study done on RAW 264.7 cells, it was found that the compound 2-(2-phenylethyl) chromone, extracted from agarwood, reduces nitric oxide (NO) production by inhibiting nuclear factor kappa-light-chain-enhancer of activated B cells (NF-κB) inflammatory pathway activation (Alamil et al., 2022[1]). In the present study, the potential of agarwood oil to suppress lipopolysaccharide (LPS)-induced inflammation in human bronchial epithelial cells was explored. In addition, the clinical use of agarwood oil is limited due to poor solubility and oral bioavailability. This problem was overcome by preparing agarwood oil nanoemulsion.

Agarwood nano-emulsion was prepared by the probe sonication method. The experiments were performed on human bronchial epithelial cell line (BEAS-2B) obtained as a gift from Woolcock Institute of Medical Research Laboratories. Cells were cultured in Dulbecco's Modified Eagle Medium (DMEM) supplemented with 10 % foetal bovine serum (FBS) (Gibco/Thermofisher) and 1 % antibiotic (Sigma/Merck) at 37 ℃ and 5 % CO_2_ in a humidified environment. Cells were seeded in 12-well plates (Nunc/Thermofisher scientific) at 5x10^4^ cells/ml and were incubated for 72 hours in DMEM with 10 % FBS. After that, cells were washed with 1 ml per well of Hanks' Balanced Salt Solution (HBSS, Sigma/Merck), incubated for another 24 hours in DMEM with 0.1 % FBS and 1 % antibiotic. The agarwood oil formulations were prepared at three concentrations (1, 10, and 20 µg/ml) in DMEM with 0.1 % FBS and 1 % antibiotic. The formulations were added to the cells at 1 ml/well after removing existing media and were incubated for 20 hours. After incubation, 10 µl of LPS (Sigma/Merck) were added to the treated cells at 1 µg/ml final concentration. Supernatants were collected four hours later and stored at -20 ℃ for further analysis.

To perform the cell viability assay, BEAS-2B cells were seeded in 96-well plates at 5x10^4^ cells/ml and were incubated for 72 hours in DMEM with 10 % FBS. After that, cells were washed with 1 ml/well of HBSS (Sigma/Merck), incubated for another 24 hours in DMEM with 0.1 % FBS and 1 % antibiotic. After the quiescence step, various doses of agarwood oil formulations were added at 100 µl per well and incubated for 20 hours followed by 4 hours of LPS at 1 µg/ml. Then, 3-(4,5-dimethylthiazol-2-yl)-2,5-diphenyltetrazolium bromide (MTT, Sigma Aldrich/Merck) (5 mg/ml stock solution) was added at 10 µl per well and further incubated for 3 hours at 37 ℃ and 5 % CO_2_ in a humidified environment. Then the cells were examined microscopically to observe the color change on viable cells, after which the media was aspirated from the cells and replaced with 100 µl per well of dimethyl sulfoxide (DMSO) (Sigma/Merck) for 5 minutes, mixed well by pipetting, and then the absorbance was measured using a spectrophotometer at a wavelength of 570 nm.

ELISA (using commercial antibody kits according to manufacturer's instructions) was performed on 96 plates. The plates were first coated with a layer of 10 µl of capture antibody (500 µg/ml) diluted into 10 ml of 0.1 M Na_2_HPO_4_. The solution was added at 100 µl per well and incubated (all incubations during ELISA were done on a shaking platform) for 24 hours at 4 ℃. After that, the plates were washed 4 times with tween phosphate-buffered saline (T-PBS) and blocked using 200 µl/well of 1 % bovine serum albumin (BSA) diluted in PBS. The plates were incubated for either 1 hour at room temperature or for 24 hours at 4 ℃. Standards were prepared by adding 10 µl of the standard solution to 990 µl of DMEM media with 0.1 % FBS and 1 % antibiotic and the other 7 standard solutions were serially diluted by adding 500 µl of media and 500 µl of the solution of higher concentration. The plates were washed 3 times with T-PBS before adding the prepared standards in duplicate and 100 µl of experimental supernatants in duplicate. The plates were incubated for 2 hours at room temperature or for 24 hours at 4 ℃. Next, the plates were washed 4 times with T-PBS. After that, a diluted 10 µl of the 500 µg/ml detection antibody into 10 ml of 1 % BSA-PBS for IL-6 and 0.1 % BSA-PBS for IL-8 was added at 100 µl per well, and then incubated for a further 1 hour at room temperature. Then, the plates were washed 6 times with T-PBS and a solution of 50 µl of streptavidin-horseradish peroxidase diluted into 10 ml of 1 % BSA-PBS for IL-6 and 0.1 % BSA-PBS for IL-8 was added at 100 µl per well and incubated for 30 minutes in the dark at room temperature. Finally, the plates were washed 8 times with T-PBS, and a 100 µl per well of 3,3',5,5'-Tetramethylbenzidine (TMB) reagent mix was added. Then, the plates were observed for color change for 2-5 minutes, and when the desired color was detected, the reaction was immediately stopped by adding 100 µl per well of 1 M phosphoric acid. The plates were then read with a spectrophotometer at 450 nm and 570 nm for reference. Statistical analysis was done using GraphPad Prism version 9.4. Two-way ANOVA test was performed to compare the untreated cells to the ones treated with agarwood oil with or without LPS.

There was no significant effect of the agarwood oil at the three different concentrations applied (1 µg/ml, 10 µg/ml, 20 µg/ml) on the viability of the BEAS-2B cells after 24 hours of treatment (p value 0.3731 of highest concentration) (figure 1). The cell viability was not affected by agarwood oil with LPS added at 1 µg/ml (p value 0.5141 of highest concentration of agarwood formulation).

Figure 2 shows that there was no significant difference on the levels of IL-6 (figure 2a) and IL-8 (figure 2b) in control BEAS-2B cells (agarwood untreated) compared to those treated with agarwood formulation for 20 hours at three concentrations (1 µg/ml, 10 µg/ml, 20 µg/ml) (figure 2). However, stimulation of BEAS-2B with 1 µg/ml of LPS for 4 hours significantly increased the levels of both IL-6 and IL-8 (figure 2a and 2b). In contrast, pre-treatment of BEAS-2B with agarwood formulation before LPS stimulation, resulted in a dose-dependent and significant decrease in the levels of both cytokines (figure 2a and 2b). 

The study showed that inflammatory effect was exerted by LPS on BEAS-2B cells as revealed by the significant increase in the levels of IL-6 and IL-8. BEAS-2B cells are healthy human bronchial epithelium derived cells and they are ideal for performing *in vitro* studies of various respiratory diseases. The cellular inflammatory response of BEAS2B cells to LPS is a model constantly adopted by researchers to study the anti-inflammatory potential of various drugs/herbal compounds (Verspohl and Podlogar, 2012[9]). Considerable studies have proven that LPS (endotoxin in gram negative bacteria) induces cellular inflammation by activating several inflammatory pathways like NF-κB that ultimately leads to the production of the cytokines IL-6, IL-8, and TNF-α (Hou et al., 2021[3]; Si and Zhang, 2021[8]). Therefore, LPS was used to stimulate the BEAS-2B cells in our study as an *in vitro* model of infection-induced airway inflammation (Mehta et al., 2020[4]; Edwards et al., 2009[2]).

Experimental results from the study also revealed that agarwood oil emulsion has a significant anti-inflammatory action in LPS-induced BEAS-2B cells. The anti-inflammatory potential of agarwood oil emulsion was evident by the observed inhibition in the LPS-induced production of IL-6 and IL-8. Based on these facts, there can be several possibilities upon which agarwood oil could have exerted its biological action, including hypothalamic adrenal pituitary axis (HPA), NF-κB, p38 mitogen-activated protein kinase (MAPK), signal transducer and activator of transcription (STAT), and Cyclooxygenase (COX) pathways.

Despite being used in different forms by people for centuries, there is not enough scientific data on its toxicity, possible allergic reactions, or safety for different patient groups (Mokhtar et al., 2021[5]). The cell viability assay of the current study in discussion showed no significant toxic effect exerted by agarwood on BEAS-2B cell viability. This study provides supportive data for the safety of agarwood emulsion at the maximum concentration of 20 µg/ml used on human bronchial epithelial cells. However, this data does not provide conclusive information on the toxicity of agarwood. Further experiments are required before agarwood becomes a clinically proven agent.

Based on previous studies on the nature and properties of agarwood extracts, there are variety of possibilities that can be put into consideration when thinking about routes of administration of agarwood. For example, a molecular docking and ADME studies done to test agarwood oil against skin inflammatory conditions were proven effective as shown by significant reduction in the inflammatory state (Yadav et al., 2013[10]). In addition, an *in vitro* study investigating the anti-inflammatory effect of agarwood incense smoke showed a significant decrease in the production of inflammatory cytokines (TNF-α and IL-1b) produced by LPS-induced mice macrophage cells (RAW264.7) (Peng et al., 2020[6]). These studies show that agarwood oil could be used in many forms to target different inflammatory diseases at various sites. This could also be tailored to overcome side effects, as well as personal preference of patients based on their age group, health state, and abilities. 

The results of the present study are very promising, however, there are some limitations. This study was done *in vitro* using human bronchial epithelial cells that are away from their natural niche which may lead to change in their behavior compared to ones in human subjects surrounded by other functioning cells. In addition, there could be variation in the data owing to the difference in the batches or samples. Future prospects of this study are to perform *in vivo* study on asthma or COPD rat models applying different methods of administration of agarwood formulation in order to assess its effectiveness as an anti-inflammatory agent as well as its possible routes of administration for further advanced studies.

## Notes

Dinesh Kumar Chellappan and Kamal Dua (Discipline of Pharmacy, Graduate School of Health, University of Technology Sydney, Sydney, NSW 2007, Australia; E-mail: Kamal.Dua@uts.edu.au) contributed equally as corresponding author.

## Conflict of interest

The authors declare no conflict of interest.

## Supplementary Material

Supplementary information
