# Supplementary material for: Agarwood oil nanoemulsion attenuates production of lipopolysaccharide (LPS)-induced proinflammatory cytokines, IL-6 and IL-8 in human bronchial epithelial cells
Source: EXCLI J. 2023 Jul 20;22:681–5. doi: 10.17179/excli2023-6282 (PMC10471838; doi:10.17179/excli2023-6282)
Supplement: Supplementary information [file EXCLI-22-681-s-001.pdf]

EXCLI Journal 2023;22:681-685 – ISSN 1611-2156
Received: June 26, 2023, accepted: July 11, 2023, published: July 20, 2023

Supplementary information to:

Letter to the editor:

AGARWOOD OIL NANOEMULSION ATTENUATES PRODUCTION OF LIPOPOLYSACCHARIDE (LPS)-INDUCED PROINFLAMMATORY CYTOKINES, IL-6 AND IL-8 IN HUMAN BRONCHIAL EPITHELIAL CELLS

Juman Mohammed Rasmi Alamil[1], Dikaia Xenaki[2], Bikash Manandhar[3,4], Keshav Raj Paudel[5], Philip Michael Hansbro[5], Brian Gregory Oliver[2,6], Dinesh Kumar Chellappan[7,*], Kamal Dua[3,4,8,*]

1 School of Postgraduate Studies, International Medical University (IMU), Kuala Lumpur 57000, Malaysia
2 Woolcock Institute of Medical Research, Macquarie University, Sydney, NSW 2137, Australia
3 Discipline of Pharmacy, Graduate School of Health, University of Technology Sydney, Sydney, NSW 2007, Australia
4 Australian Research Center in Complementary and Integrative Medicine, Faculty of Health, University of Technology Sydney, Ultimo, NSW 2007, Australia
5 Center for Inflammation, Centenary Institute and University of Technology Sydney, Faculty of Science, School of Life Sciences, Sydney, NSW 2050, Australia
6 School of Life Sciences, Faculty of Science, University of Technology Sydney, Ultimo, NSW 2007, Australia
7 Department of Life Sciences, School of Pharmacy, International Medical University, Bukit Jalil, Kuala Lumpur 57000, Malaysia
8 Uttaranchal Institute of Pharmaceutical Sciences, Uttaranchal University, Dehradun 248007, India

* **Corresponding authors:** Dr. Kamal Dua, Discipline of Pharmacy, Graduate School of Health, University of Technology Sydney, Sydney, NSW 2007, Australia.
E-mail: Kamal.Dua@uts.edu.au
Dr. Dinesh Kumar Chellappan, Department of Life Sciences, School of Pharmacy, International Medical University, Bukit Jalil, Kuala Lumpur 57000, Malaysia.
E-mail: Dinesh_Kumar@imu.edu.my

https://dx.doi.org/10.17179/excli2023-6282

This is an Open Access article distributed under the terms of the Creative Commons Attribution License (http://creativecommons.org/licenses/by/4.0/).

Received: June 26, 2023, accepted: July 11, 2023, published: July 20, 2023

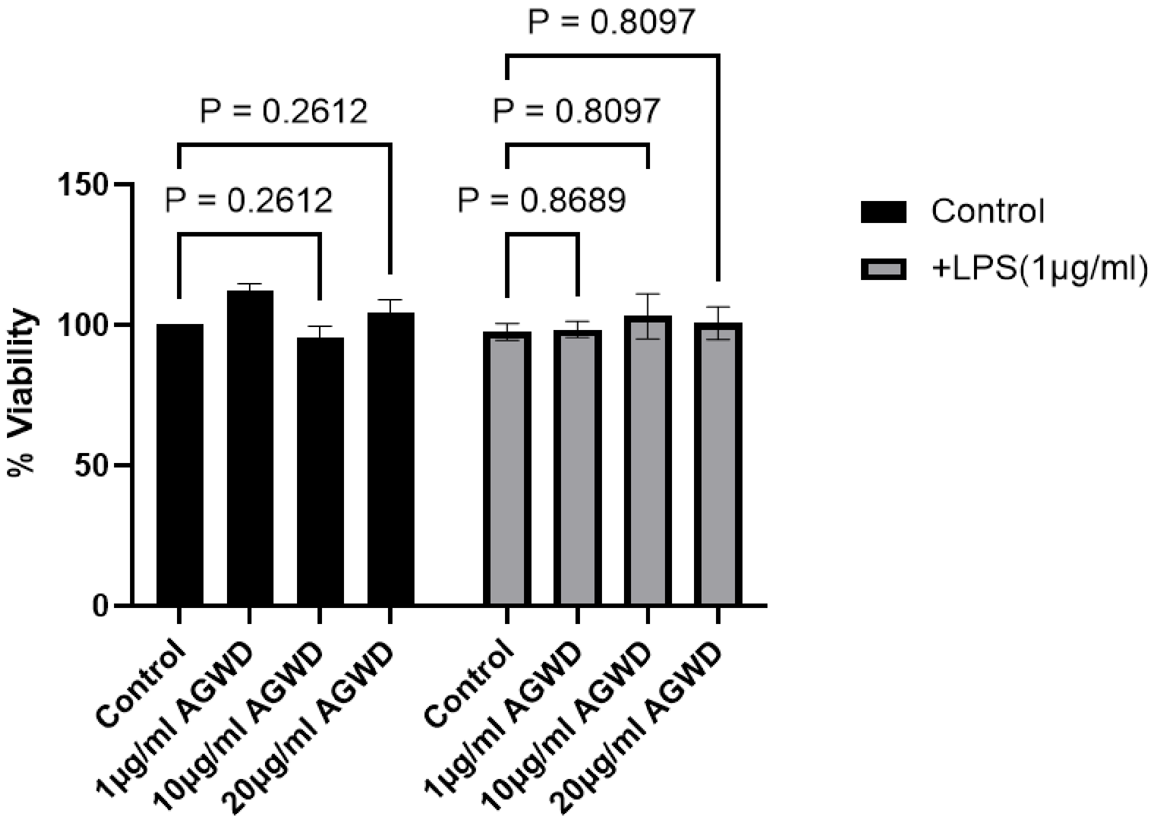

Supplementary Figure 1: The effect of agarwood formulation on cell viability. Cell viability was assessed 24 hours after treatment by MTT colorimetric assay by measuring the absorbance of formazan using spectrophotometer at 540 nm. Data was analyzed with two-way ANOVA as well as multiple comparison study at n=2. Cells treated with agarwood formulation and those treated with agarwood formulation in addition to LPS were compared to control with no significance found $p > 0.05$.

AGWD: agarwood formulation, LPS: lipopolysaccharide

EXCLI Journal 2023;22:681-685 – ISSN 1611-2156
Received: June 26, 2023, accepted: July 11, 2023, published: July 20, 2023

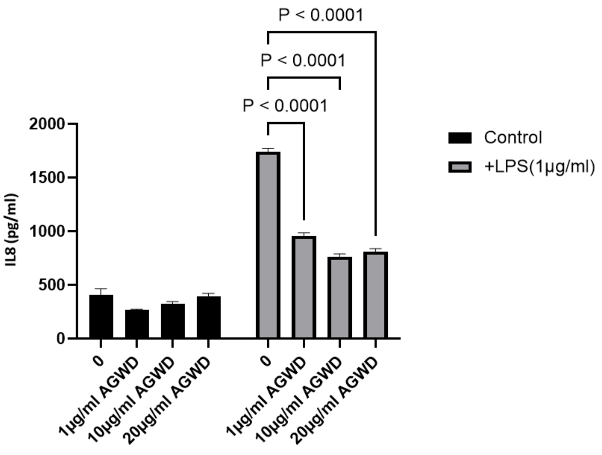

Figure 2 (b)
IL-8

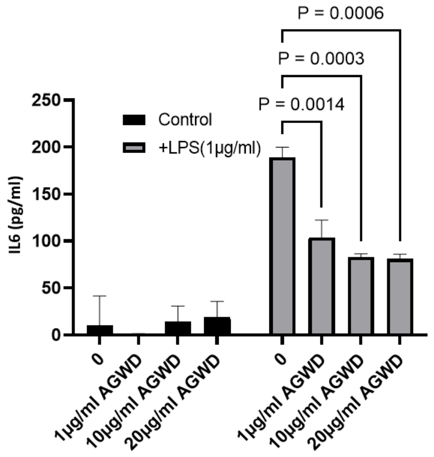

Figure 2 (a)
IL-6

Supplementary Figure 2: Effect of agarwood oil on (a) IL-6 and (b) IL-8 production in LPS induced BEAS-2B cells. The level of (**2a**) IL-6 and (**2b**) IL-8 in the LPS and/or agarwood formulation treated BEAS-2B cell culture supernatants was quantified by ELISA. Statistical analysis was then done by two-way ANOVA as well as multiple comparison test at n=12 showing statistical significance $p<0.0001$.

AGWD: agarwood formulation
